# Stopover optimization in a long-distance migrant: the role of fuel load and nocturnal take-off time in Alaskan northern wheatears (*Oenanthe oenanthe*)

**DOI:** 10.1186/1742-9994-10-26

**Published:** 2013-05-12

**Authors:** Heiko Schmaljohann, Fränzi Korner-Nievergelt, Beat Naef-Daenzer, Rolf Nagel, Ivan Maggini, Marc Bulte, Franz Bairlein

**Affiliations:** 1 Institute of Avian Research “Vogelwarte Helgoland”, An der Vogelwarte 21, Wilhelmshaven 26836, Germany; 2 Swiss Ornithological Institute, Seerose 1, Sempach, 6204, Switzerland; 3 Oikostat GmbH, Ausserdorf 43, Ettiswil, 6218, Switzerland; 4 Current Address: Advanced Facility for Avian Research, University of Western Ontario, 1151 Richmond Street, London, ON, N6G 1G9, Canada

**Keywords:** Arctic, Capture-recapture, Cormack-Jolly-Seber model, Departure probability, Departure time, Migration speed, Northern wheatear, Optimization, Songbird, Stopover ecology

## Abstract

**Introduction:**

In long-distance migrants, a considerably higher proportion of time and energy is allocated to stopovers rather than to flights. Stopover duration and departure decisions affect consequently subsequent flight stages and overall speed of migration. In Arctic nocturnal songbird migrants the trade-off between a relatively long migration distance and short nights available for travelling may impose a significant time pressure on migrants. Therefore, we hypothesize that Alaskan northern wheatears (*Oenanthe oenanthe*) use a time-minimizing migration strategy to reach their African wintering area 15,000 km away.

**Results:**

We estimated the factors influencing the birds’ daily departure probability from an Arctic stopover before crossing the Bering Strait by using a Cormack-Jolly-Seber model. To identify in which direction and when migration was resumed departing birds were radio-tracked. Here we show that Alaskan northern wheatears did not behave as strict time minimizers, because their departure fuel load was unrelated to fuel deposition rate. All birds departed with more fuel load than necessary for the sea crossing. Departure probability increased with stopover duration, evening fuel load and decreasing temperature. Birds took-off towards southwest and hence, followed in general the constant magnetic and geographic course but not the alternative great circle route. Nocturnal departure times were concentrated immediately after sunset.

**Conclusion:**

Although birds did not behave like time-minimizers in respect of the optimal migration strategies their surplus of fuel load clearly contradicted an energy saving strategy in terms of the minimization of overall energy cost of transport. The observed low variation in nocturnal take-off time in relation to local night length compared to similar studies in the temperate zone revealed that migrants have an innate ability to respond to changes in the external cue of night length. Likely, birds maximized their potential nightly flight range by taking off early in the night which in turn maximizes their overall migration speed. Hence, nocturnal departure time may be a crucial parameter shaping the speed of migration indicating the significance of its integration in future migration models.

## Introduction

Long-distance migrations in songbirds are covered by migratory flights and intermittent resting and re-fuelling phases (stopovers). Only a minor proportion of time and energy is allocated to flight stages [1-3], whereas fuelling during stopover is a demanding and slow process. Hence, stopover behaviour is of major significance for optimizing migration in terms of energy and time costs [4]. How Arctic migrants adjust their stopover strategies to the relatively long migration-distances and the short nights in the Arctic is largely unknown [5].

**Figure 1:**
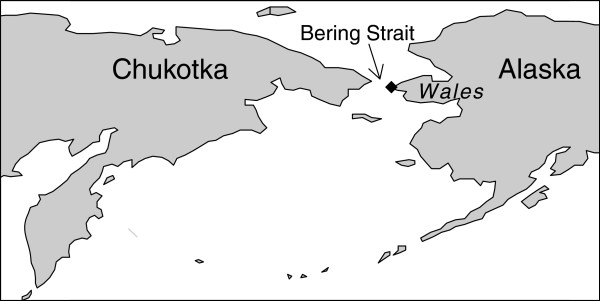
**Location of the study site Wales in Alaska. **From there about 80 km are to be covered across the Bering Strait to reach the Russian mainland.

Here we assessed departure rules for an extreme long-distance migratory songbird, the Alaskan northern wheatear, *Oenanthe oenanthe* (hereafter wheatear). We studied its stopover ecology at a coastal stopover site in western Alaska (Figure 1) prior to a nearly 15,000 km migration across Asia to eastern Africa [6]. Specifically, we determined fuel deposition rate and departure fuel load by using baited electronic balances and departure time and direction through radio transmitters. Considering the extraordinary migration distance and earlier evidence about optimal migration strategy mostly along the European flyway in songbirds [5,7-10], we hypothesize that Alaskan wheatears behave like time-minimizers at the Arctic stopover site [1,4]. Individual departure decisions depend on intrinsic factors, such as fuel load and fuel deposition rate, and environmental cues, such as resource availability and meteorological conditions [11-14]. We, therefore, expect that the probability of departure increases with stopover duration, fuel load and wind support.

In Arctic nocturnal migrants, the short duration of the night may impose a serious constraint in optimising migratory flight stages. As more time is spent on the ground than flying [3] the total number of stopovers significantly contributes to the overall speed and costs of migration [1,4]. Actually, we lack any information on whether birds adjust the timing of their nocturnal departure in respect of the duration of the night and/or in respect of the migratory distance to be covered. Earlier studies demonstrated a large variation in nocturnal departure times [14-24], but see [25]. Considering the long migration distance of Alaskan wheatears and their high total migration speed of 160 km day^-1^[3] in comparison to European wheatears (4,000 km and 88 km day^-1^; [26]) we hypothesize that Alaskan wheatears fully exploit the available night-time for migration. Doing so would help explain the extreme long nocturnal travel ranges of on average 330 km night^-1^ found in Alaskan wheatears during autumn migration [3].

**Figure 2:**
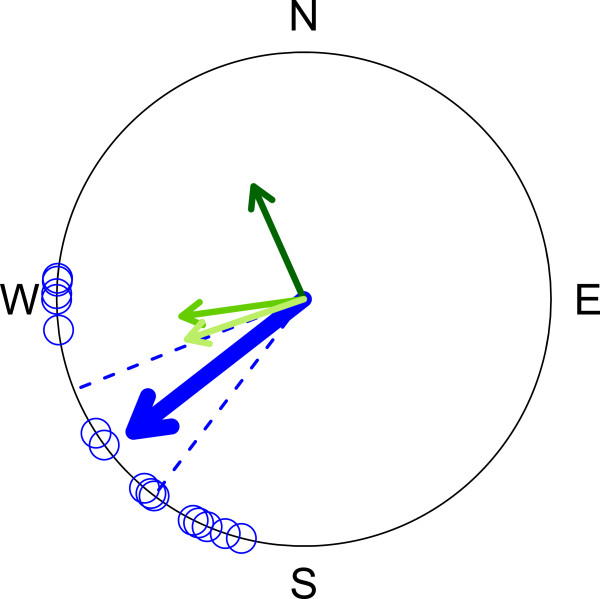
**Departure directions of northern wheatears from Wales, Alaska. **Departure directions were uniformly distributed towards 233° (Rayleigh test of uniformity: *n *= 15, *R *= 0.87, *P *< 0.0001; 95% CI: 218° – 249°, range: [205°, 288°]). The blue arrow shows the mean departure direction and its length being a measure of scatter is drawn relative to circle’s radius which is one (unit circle). Dashed lines give 95% confidence interval. Equal departure directions were plotted at slightly different directions so that they can be recognized (Table 1). The short arrows indicate the directions of the great circle route (336°, dark green), the constant magnetic course (262°, middle green) and the constant geographic course (251°, light green) from the study site towards Alaskan northern wheatears’ wintering area in sub-Saharan Eastern Africa.

As shown for Alaskan wheatears tracked with light-level geolocators [3], we expect that wheatears leave our study site towards the West (constant magnetic and geographic course, Figures 1 and 2) and do not follow the predicted great circle route [27]. Identifying the decisions why, when and to where Alaskan wheatears resume migration is the first step in understanding their movement ecology [28].

## Materials and methods

The study site was located at the westernmost point of mainland Alaska in Wales (65°37′N, 168°5′W, USA; Figure 1). From there about 80 km are to be covered across the Bering Strait to reach the Russian mainland, which is visible in clear conditions. The small Diomede Islands lie halfway through (45 km).

### Fuel loads and fuel deposition rate

Wheatears were caught with spring traps, banded and colour ringed. Body mass at capture, i.e., arrival, was measured to the nearest 0.1 g, wing length to the nearest 0.5 mm and age ascertained as previously described [29].

Birds’ body mass change during stopover was measured using baited electronic balances. This is so far the only practical approach to weigh free-ranging songbirds repeatedly and shortly before departure [5,7-10,30-37]. Six bowls, at a distance of 5 to 15 m from each other, were supplied with mealworms, *Tenebrio molitor*, throughout the daylight period from 11^th^ to 31^st^ of August 2010. Three electronic balances (WEDO Digi 2000, Münster, Germany) were placed alternatingly underneath the bowls. By using telescopes the weight of wheatears visiting the bowls was determined to the nearest 0.1 g. The last weight within 2 hours before sunset was denoted the evening body mass of that day for the bird. If the bird carried a transmitter, the mass was corrected accordingly. Fuel load was estimated as previously described [35]:

(1) $$\begin{array}{ll} {\text{arrival}\;\text{fuel}\;\text{load}}_{i} & =\left({\text{arrival}\;\text{body}\;\text{mass}}_{i}\left[g\right]\right)\\ & \;–\left({\text{lean}\;\text{body}\;\text{mass}}_{i}\left[g\right]\right)/{\text{lean}\;\text{body}\;\text{mass}}_{i}\left[g\right], \end{array}$$

(2) $$\begin{array}{ll} {\text{evening}\;\text{fuel}\;\text{load}}_{i} & =\left({\text{evening}\;\text{body}\;\text{mass}}_{i}\left[g\right]\right)\\ & \;–\left({\text{lean}\;\text{body}\;\text{mass}}_{i}\left[g\right]\right)/{\text{lean}\;\text{body}\;\text{mass}}_{i}\left[g\right] \end{array}$$

with

(3) $$\begin{array}{ll} {\text{lean}\;\text{body}\;\text{mass}}_{i}\left[g\right] & =0.{29\;g\;\mathrm{mm}}^{-1}\\ & \;\times {\mathrm{wing}\;\mathrm{length}}_{i}\left[\mathrm{mm}\right]–6.85\;g \end{array}$$

as previously described [14]. Evening fuel load calculated for the last day of stopover was treated as departure fuel load. Total fuel deposition rate was estimated as previously described [35], but using arrival body mass instead of first evening body mass:

(4) $$\begin{array}{ll} {\text{fuel}\;\text{deposition}\;\text{rate}}_{i}\left[d^{-1}\right] & =\left({\text{departure}\;\text{fuel}\;\text{load}}_{i}\left[g\right]\right)\\ & \;–\left({\text{arrival}\;\text{body}\;\text{mass}}_{i}\left[g\right]\right)/{\text{lean}\;\text{body}\;\text{mass}}_{i}\\ & \;\left[g\right]/{\text{minimum}\;\text{stopover}\;\text{duration}}_{i}\left[d\right]\text{.} \end{array}$$

We used wheatears’ fuel deposition rate and departure fuel load to discern their optimal migration strategy (Additional file 1).

### Minimum stopover duration

We considered the minimum stopover duration as the number of days spent in the study area, including the day of trapping, based on daily searches for colour-ringed wheatears. For individuals not re-encountered after ringing we did not estimate the minimum stopover duration, as they were transients at our site [38]. We defined the re-sighting probability of an individual in the field as the number of days with observations divided by its minimum stopover duration. The corresponding re-sighting probability in the field was 0.96 (*n* = 40). The minimum stopover duration was 4.3 ± 1.6 days (max. 8 days). Departure body mass could be identified for 21 out of those 40 wheatears. Their average re-sighting probability was 0.99 and their minimum stopover duration 4.5 ± 1.5 days. This is compared to stopover duration estimations modelled on the basis of individual re-encounter histories by a Cormack-Jolly-Seber model, details of the model and comparisons are given below (see *Mark-recapture model*).

### Radio tracking

Wheatears were tracked using radio transmitters of own construction [39]. The transmitters were tuned to maximum radiated power (range) and had a life of c. 30 d. Including harness the tags weighed 0.8 g. They were attached using a Rappole-Tipton-type harness made from 0.5 mm elastic cord [40]. Length of leg-loops was adjusted individually [41]. As the minimum body mass of tagged birds was 24.2 g, radio transmitter load represented < 3.3% of birds’ body mass and was below the recommended 5% limit [42-45].

We used Yagi 3EL2 hand-held antennas (Vårgårda, Sweden) in combination with FT-290RII receivers (Yaesu, Japan). The detection range of the radio transmitters was 12 to 15 km [14]. Every night, except on the 14^th^ and 15^th^ of August (severe weather conditions with strong gale, wind gust with 90 km/h^-1^ and heavy rain preventing bird migration [46]), we tracked birds continuously from sunset till early morning or until departure. Radio-tracking made it easy to detect when the birds left the mainland and headed to the open sea. Departing birds were tracked until loss of signal. According to the bearings, birds departed in a straight line from the coast. We used the last recorded direction before loss of signal as the departure direction using a compass (Additional file 2). The bearings were corrected for the local declination (+11.19°; derived via http://www.ngdc.noaa.gov/geomag-web/#declination on 14^th^ March 2012) at Wales on 15^th^ of August 2010.

Seven of the 30 radio-tagged individuals had left the study area on the day of trapping (transients) or during the subsequent day but not during the night (Table 1). Signals of another seven birds were lost during the night: In the early night their corresponding signals were located in the mountains suggesting that they rested there. During the nights their signals vanished. If these birds had set off, signal strength would first have increased strongly and then continually decreased for the next c. 15 min. As we did not observe this typical pattern in signal strength prior to the disappearance of the birds, we denoted these birds as lost, though we assume that they did not depart at that night (Table 1; [15]).

**Table 1:** Radio tracking details of northern wheatears in Wales (AK) in autumn 2010

**Ind. [ring number]**	**Date of capture**	**Night of departure**	**Departure time [min after local sunset]**	**Sun elevation at departure [°]**	**Departure direction [°]**	**Tracking information**
179106207	11.8.	-	-	-	-	- loss of signal during the night on 12./13.8.
						- tracked until 40 min after sunset*
179106208	11.8.	18/19.8.	83	-6.68	219	
179106210	12.8.	18/19.8.	103	-7.87	194	
179106211	12.8.	12/13.8.	85	-6.17	201	
179106212	12.8.	17/18.8.	114	-8.33	204	
179106214	12.8.	17/18.8.	124	-8.80	209	
179106215	13.8.	-	-	-	-	- loss of signal during the night on 13./14.8.
						- tracked until 305 min after sunset*
179106224	14.8.	18/19.8.	68	-5.70	239	
179106229	14.8.	-	-	-	-	- loss of signal during the night on 20./21.8.
						- tracked until 165 min after sunset*
179106236	14.8.	-	-	-	-	- no tracking during the night of 14./15.8. due to strong gale and rain - likely disappeared during the day of 14.8. (transient)
179106242	15.8.	18/19.8.	128	-9.14	219	
179106257	19.8.	-	-	-	-	- loss of signal during the night on 19./20.8.
						- tracked until 220 min after sunset*
179106259	19.8.	-	-	-	-	- disappeared during the day
						- tracked until the morning of 20.8., but not relocated during the day
179106261	20.8.	20/21.8.	76	-6.37	219	
179106262	20.8.	26/27.8.	95	-8.11	277	
179106263	21.8.	-	-	-	-	- loss of signal during the night on 21./22.8.
						- tracked until 270 min after sunset*
179106264	21.8.	26/27.8.	55	-5.49	264	
179106265	22.8.	26/27.8.	99	-8.47	274	
179106266	22.8.	25/26.8.	29	-3.47	-	- departure direction could not be determined
179106271	22.8.	-	-	-	-	- disappeared during the night on 22./23.8.
						- tracked until 195 min after sunset*
179106275	22.8.	-	-	-	-	- disappeared during the night on 22./23.8.
						- tracked until 165 min after sunset*
179106276	22.8.	26/27.8.	48	-4.92	269	
179106277	23.8.	26/27.8.	114	-9.45	274	
179106286	26.8.	-	-	-	-	- disappeared during the day (transient)
						- not relocated on the evening of 26.8.
179106297	29.8.	-	-	-	-	- disappeared during the day (transient)
						- not relocated on the evening of 29.8.
179106298	29.8.	29/30.8.	87	-7.90	204	
179106299	29.8.	-	-	-	-	- disappeared during the day (transient)
						- not relocated on the evening of 29.8.
181108935	29.8.	29/30.8.	54	-5.60	234	
181108944	31.8.	-	-	-	-	- disappeared during the day (transient)
						- not relocated on the evening of 31.8.
181108945	31.8.	-	-	-	-	- disappeared during the day (transient)
						- not relocated on the evening of 31.8.

Departure time was defined as time when birds actually set off from the ground, as signal strength increases dramatically due to good reception of the signal. Departure direction was corrected for local declination (+11.19°, see *Methods* section). If departure could not be tracked, circumstances of last tracking are given. Even after loss of signal birds’ specific frequencies were checked throughout the night to detect whether birds reappeared or departed. Birds leaving the study area before the evening of their day of capture were transients. * = Bird rested in stony mountain. Signal vanished without prior increase in signal strength.

Wheatears were caught and radio-tagged under licence of the U.S. Fish and Wildlife Service (Federal Fish and Wildlife Permit: MB207892-0).

### Compass courses

Predicted migration directions from the study site to the known wintering area of Alaskan wheatears in sub-Saharan eastern Africa (34°E, 8°N) was estimated for (1) the great circle route [27,47,48] (336°, sun compass course not compensating for the change in local time during migration), (2) the constant geographic course [49] (251°, star and sun compass course compensating for the change in local time, i.e., rhumb line) and (3) the constant magnetic course [49] (262° for the first 10 km of wheatears’ migration off Wales, as previously described [3]).

### Meteorological data

Sun’s elevation, time of sunset and time of sunrise were derived from http://www.esrl.noaa.gov/gmd/grad/solcalc/ on 14^th^ and 22^nd^ of March 2012. To estimate the effect of temperature and wind on wheatears’ departure decision, we considered NCEP/DOE Reanalysis II data from the National Oceanic and Atmospheric Administration (NOAA, Boulder, CO, USA; available http://www.cdc.noaa.gov/cdc/data.ncep.reanalysis.derived.html; [50]). Wind data were obtained via the R-package *RNCEP*[51] for surface and four different pressure levels (1000, 925, 850 and 700 mbar; only wind data). Data were interpolated in respect to our study site and local midnight for the whole study period [51], because general departure time of wheatears was nearest to local midnight (Table 1).

We used tailwind component to consider the effect of wind on birds’ flight:

(5) $$\begin{array}{l} {\text{tailwind}\;\text{component}}_{i}\left[{m\;s}^{-1}\right]\\ \;=\text{cos}\left({\text{wind}\;\text{direction}}_{i}–\text{migratory}\;\text{goal}\right)\\ \;\times {\text{wind}\;\text{speed}}_{i}\left[{m\;s}^{-1}\right]\text{.} \end{array}$$

However, estimates of wind profit [52] are a better approach to quantifying wind support, but wind speed was often too high to estimate wind profit. We considered maximum wind tailwind component of the five different pressure levels (see above), because flapping and bounding flyers select altitude with best wind support [53]. Individual tailwind component was estimated for bird’s departure time within the night [51]. To simplify matters we assumed the flight direction to the next migratory goal to be 233° (Figure 2) or to the individually tracked departure direction.

### Flight range

Birds’ potential flight duration is a function of fuel load [9]:

(6) $${\text{flight}\;\text{duration}}_{i}\left[h\right]=100\times \ln\left(1+{\text{fuel}\;\text{load}}_{i}\right)\text{.}$$

As outlined above, the flight duration in nocturnal migrants is not only restricted by fuel load, but also by the time available between departure and sunrise. To estimate the bird’s potential time for its nocturnal migratory flight until sunrise, we considered the birds’ individual departure time after sunset if radio-tracked or the mean departure time after sunset (85 min) and bird’s maximum tailwind component:

(7) $$\begin{array}{ll} {\text{nocturnal}\;\text{flight}\;\text{range}}_{i}\left[\mathrm{km}\right] & ={\text{restricted}\;\text{flight}\;\text{range}}_{i}\left[\mathrm{km}\right]\\ & \;+{\text{nocturnal}\;\text{flight}\;\text{duration}}_{i}\left[h\right]\\ & \;\times {\;\text{tailwind}\;\text{component}}_{i}\left[{\mathrm{km}\;h}^{-1}\right]\text{.} \end{array}$$

We estimated the distance to the nearest land in respect of the birds’ departure directions by considering the nearest land within 7.5° around the departure direction (Google Earth 6.2).

### Mark-recapture model for estimating departure probability and stopover duration

a) Departure probability

We used a state-space formulation of a parameter constrained Cormack-Jolly-Seber model [54-56] to estimate the daily apparent survival probability *Φ*_*i,t*_ which is the probability that an individual survives to the next day and stays in the area. Because the probability to survive 24 hours is in our case close to one, *Φ*_*i,t*_ can be interpreted as staging probability, which is 1 - departure probability. The binary latent state variable *z*_*i,t*_ described whether an individual was present in the study area (*z*_*i,t*_ = 1) or not (*z*_*i,t*_ = 0). The state of individual *i* at day *t* was modelled as a Bernoulli process depending on the state at day *t*-1 and the staging probability *Φ*_*i,t*_*: z*_*i,t*_ ~ Bernoulli(*z*_*i,t-1*_*Φ*_*i,t-1*_).

The probability that a bird was observed during one day (detection probability) was modelled as a Bernoulli process: an individual *i* was observed at day *t* with the probability *z*_*i,t*_*p*_*i,t*_: *Y*_*i,t*_ ~ Bernoulli(*z*_*i,t*_*p*_*i,t*_), where *z*_*i,t*_ is an indicator variable indicating whether individual *i* is present at the study site at day *t* and *p*_*i,t*_ is the detection probability. The logit of detection probability was constrained to be linearly dependent on an indicator variable for radio-tagged individual (*radio-tagged*) and on surface wind speed (standardised, *wind.z*). As birds may react to strong wind conditions with a more sheltered behaviour, wind speed may decrease birds’ detection probability for both colour marked and radio-tagged individuals.

(8) $$\text{logit}\left(p_{i,t}\right)\sim \beta_{1}+\beta_{2}\text{xradio}-\text{tagge}d_{i}+\beta_{3}\text{xwind}.z_{t}\text{.}$$

Staging probability was predicted by day since bird’s arrival (including arrival day, *daysa*), day since start of study (centered, *day.c*), surface temperature (standardised, *temp.z*), surface wind speed (stanxdardised, *wind.z*), tail wind component (standardised, *twc.z*), arrival fuel load (standardised, *afl.z*), the interaction of arrival fuel load x day since start of study (*afl.z x day.c*) and an index variable indicating the day of arrival (*indfirst*). With this index we accounted for individuals that disappeared from our study area shortly after first capture on that day and behaved as transients [38]. Evening fuel loads (*efl*) could be estimated for 30 out of all wheatears (*n* = 105). For a specific model evening fuel load (*efl*) was considered instead of arrival fuel load. We could not include individual daily fuel deposition rates, as sample size was too low. We constrained the parameters *Φ*_*i,t*_ to be linearly dependent on covariates using the logit-link function.

In the first step, we fitted the model to the encounter history for the 75 wheatears without evening fuel load estimates.

(9) $$\begin{array}{ll} \text{logit}\left(\Phi_{i,t}\right) & =\alpha_{1}+\alpha_{2}\text{xdays}a_{i,t}+\alpha_{3}\text{xday}.c_{t}+\alpha_{4}\text{xafl}.z_{i}\\ & \;+\alpha_{5}\text{xtemp}.z_{t}+\alpha_{6}x\;\text{wind}.z_{t}+\alpha_{7}\text{xtwc}.z_{t}\\ & \;+\alpha_{8}\text{xindfirs}t_{i,t}+\alpha_{9}\text{xafl}.z_{i}\text{xday}.c_{t}\text{.} \end{array}$$

In the second step, the same model was fitted to the 30 individuals with evening fuel load so that arrival fuel load was replaced by evening fuel load.

(10) $$\begin{array}{ll} \text{logit}\left(\Phi_{i,t}\right) & =\alpha_{1}+\alpha_{2}\text{xdays}a_{i,t}+\alpha_{3}\text{xday}.c_{t}+\alpha_{4}\text{xtemp}.z_{t}\\ & \;+\alpha_{5}\text{xwind}.z_{t}+\alpha_{6}\text{xtwc}.z_{t}+\alpha_{7}\text{xindfirs}t_{i,t}\\ & \;+\alpha_{8}\text{xef}l_{i,t}+\alpha_{9}\mathrm{ef}l_{i,t}\text{xday}.c_{t}\text{.} \end{array}$$

We combined the information in the data analysed in the first step (*n* = 75) with the information in the data analysed in the second step (*n* = 30) for those model parameters where the corresponding variables were measured in all 105 individuals. To do so, we used the posterior distribution of the model parameters from the first step as informative priors for the corresponding parameters (coefficients for *daysa*, *day.c*, *temp.z*, *wind.z*, *twc.z*, *indfirst*) in the second step. Using informative priors is equivalent to using additional data [57]. This allowed us fitting the different parameters in the second step to different sample sizes. Thus, the parameters *α*_*1-7*_ were estimated based on data of 105 birds, whereas the parameters *α*_*8*_ and *α*_*9*_ were estimated based on data of 30 individuals (Tables 1 and 2). We could not measure for each of the 30 birds each evening fuel load. Missing values were imputed by using a linear mixed regression (Additional files 3 and 4). We applied Markov chain Monte Carlo simulations performed in WinBUGS to fit the CJS-models (Additional file 5; [58]).

**Table 2:** Estimating staging and detection probabilities of northern wheatears

**Predictors**	**Mean**	**2.5%**	**97.5%**	**Rhat**	**n.eff**
Staging probability					
*intercept (α*_*1*_*)*	0.120	-2.071	2.527	1.002	3500
*daysa (α*_*2*_*)*	-0.105	-0.766	0.536	1.002	2700
*day.c*_*t*_*(α*_*3*_*)*	-0.215	-0.484	0.014	1.002	2300
*afl.z*_*i*_*(α*_*4*_*)*	-0.173	-1.183	0.724	1.001	6200
*temp.z*_*t*_*(α*_*5*_*)*	-0.096	-1.288	1.264	1.002	1600
*wind.z*_*t*_*(α*_*6*_*)*	-0.205	-1.101	0.674	1.002	2100
*twc.z*_*t*_*(α*_*7*_*)*	0.178	-0.746	1.139	1.002	2700
***indfirst***_***i,t ***_***(α***_***8***_***)***	**-2.892**	**-5.374**	**-0.681**	**1.002**	**2300**
***afl.z***_***i ***_***x day.c***_***t ***_***(α***_***9***_***)***	**-0.225**	**-0.162**	**-0.063**	**1.001**	**5500**
Detection probability					
*Intercept (β*_*1*_*)*	2.133	-0.229	5.295	1.001	3800
***radio-tagged***_***i ***_***(β***_***2***_***)***	**27.680**	**3.874**	**71.780**	**1.001**	**22000**
***surfwind.z***_***t+1 ***_***(β***_***3***_***)***	**-1.712**	**-3.535**	**-0.3778**	**1.001**	**8900**
deviance	21.891	15.030	37.360	1.001	4100

Parameter estimates of factors influencing the staging probability *Φ* (the probability that a bird survives 24 hours and remains at the stopover site, *α*-values) and the detection probability p (the probability of detecting an individual wheatear during one day, *β*-values) for the first CJS-model fitted to data of 75 individuals. Significant effects, i.e., 95% credible interval does not include 0, are given in bold. Given are the mean, the 2.5% and 97.5% quantiles of the posterior distributions. Rhat values larger than 1.01 indicate non-convergence [59]. Parameters estimating the staging probability: *daysa *= day since arrival; *day.c *= day since start of study (centered); *afl.z* = arrival fuel load (standardized); *temp.z *= surface temperature (standardized); *wind.z *= surface wind speed (standardized); *twc.z* = tail wind component (standardized); *indfirst *= index variable indicating the day of arrival, with that variable we could account for individuals that disappeared from our study site shortly after capture and behaved as transients; *afl.z x day.c *= interaction of arrival fuel load and day since start of study. Parameters estimating the detection probability: *radio-tagged *= bird with radio transmitter; *surfwind.z *= surface wind speed (standardized).

b) Average stopover duration

To estimate bird’s stopover duration we adapted a previously described method [60]. The departure probability (1 - staging probability) was considered constant after the last observation of an individual. In our case, this assumption was not realistic, since departure probability increased with day since arrival and with evening fuel load, and the latter increased with day since arrival. We, therefore, predicted departure probability for each individual for each day after its last observation and used a Monte Carlo simulation to obtain the stopover duration of each individual (Additional file 6).

This mark-recapture model estimated the average stopover duration on the basis of the 30 individuals, for which evening fuel load was available, as 4.4 ± 0.8 days (mean ± standard error; 95% CrI: 3.0–5.9). For the same individuals the minimum stopover duration, as observed in the field, was 4.4 ± 1.5 days (see above). We, therefore, used the departure fuel loads and fuel deposition rates as observed in the field for the analysis of the optimal migration strategies.

### Other statistics

CI indicates confidence interval and CrI credible interval. We used a Rayleigh test including all departure directions to assess the significance of the mean resultant length [61,62]. Circular–linear correlations were calculated following the previously described method [62]. The p-value for a circular–linear correlation was approximated by a randomization test (*n* = 10000) [63]. In parametric tests residual analyses did not show any serious deviation from normal distribution. If not otherwise stated, mean ± standard deviations are given.

## Results

### Fuel load and fuel deposition rate

All 105 trapped wheatears were first-year birds. Abundance of birds changed considerably over the season, as periods with many birds alternated periods with few birds (Additional file 7). Average arrival fuel load (0.21 ± 0.09) was equivalent to a potential flight duration of 18.4 ± 7.5 h and to a potential flight distance of 862 ± 352 km (*n* = 105). All arriving birds carried sufficient fuel reserves for crossing the Bering Strait. More data about the condition of birds at first capture including fat and muscle score are given in the Additional file 8.

**Figure 3:**
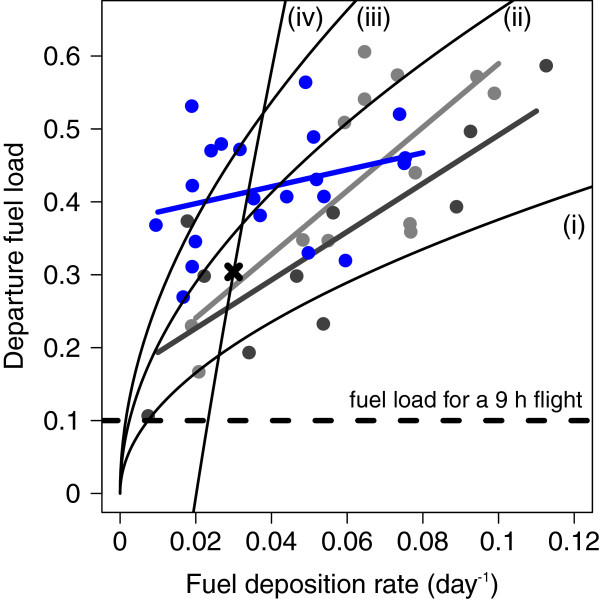
**Relationship between departure fuel load and total fuel deposition rate of northern wheatears in Wales, Alaska. **Departure fuel load and total fuel deposition rate did not correlate significantly (blue dots and blue trend line; *n *= 21; *R*_*S *_= 0.3, 95% CI = -0.22 – 0.65). Black dashed line indicates departure fuel load to accomplish a 9 h flight, which is the maximum night length experienced during the study period. If birds had minimized the overall energy costs of transport, data points should have scattered around this line [1]. For comparison we give departure fuel loads and fuel deposition rates of wheatears departing from Heimaey, Iceland (light grey dots and light grey trend line; *n *= 10, *R*_*S *_= 0.81, 95% CI = 0.16 – 1.0) [9], and from Helgoland, Germany (dark grey dots and dark grey trend line; *n* = 13, *R*_*S*_ = 0.61, 95% CI = 0.003 – 0.91) [8] during autumn. Correlation coefficients did not differ significantly from each other (compare 95% CI and Steiger’s Z-tests: *z-score *< 1.93, *P > *0.054). Black curves i – iii are the predicted relationships for time-minimizers assuming global variation with search and settling time of one day (i), three days (ii), and five days (iii). Curve iv is the predicted relationship assuming local variation as previously described [64], with recapture on Helgoland without supplement food (black cross: total fuel deposition rate = 0.030 day^-1^, departure fuel load = 0.304). There were no recaptures without supplement food in Wales.

Evening body mass on the night of departure could be determined for 21 wheatears. Their departure fuel load (0.42 ± 0.08) was significantly higher than their arrival fuel load (0.25 ± 0.09; Wilcoxon signed pair test: *n* =21, *V* = 231, *p* < 0.0001) yielding an increase in their potential flight duration from 22 ± 6.8 h to 35 ± 5.5 h and flight distance from 1050 ± 316 km to 1637 ± 259 km. The average fuel deposition rate of these birds was 0.04 ± 0.02 day^-1^ which decreased with increasing stopover duration (*n* = 21, *R*_*S*_ = -0.52, *p* = 0.015). Per stopover day wheatears increased their potential flight duration by 3 ± 1.5 h. The correlation between departure fuel load and fuel deposition rate was low (*n* = 21, *R*_*S*_ = 0.26, 95% CI = -0.22 – 0.65) indicating that this relationship corresponds to an energy minimizing strategy (Figure 3 and Additional file 1). Neither parameter correlate with surface temperature (Wilcoxon-tests: *n* = 21, *P-values >* 0.12).

### Departure decisions

Results of the mark-recapture model (first step, equation 9) showed the presence of transients (*indfirst*), i.e., the general local emigration probability was higher on the first day than later (Table 2). The daily detection probability was higher (close to 1) in radio-tagged wheatears than in colour-ringed birds and decreased with an increase in surface wind speed (*wind.z*) (Table 2).

**Figure 4:**
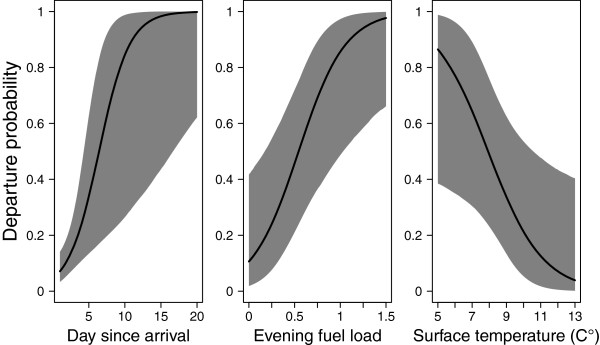
**Effect of day since arrival, evening fuel load and surface temperature on departure probability. **Departure probability is 1 - staging probability. In respect of evening fuel load and surface temperature results are shown for day five since arrival. Bold lines indicate the regression lines with the corresponding 95% CrI (grey area).

Results of the second model (considering 30 wheatears for estimating the effect of evening fuel load and all 105 for estimating the other effects, equation 10) demonstrated clearly that birds’ departure probability increased with the number of days since arrival (*daysa*), being approximately 1 after day 15 (Figure 4). As in the first model, transients seem to be present in the second model, though their effect was not significant (Table 3). Departure probability increased with evening fuel load (*efl*), e.g., about 4 out of 5 birds with an evening fuel load of 1.0 left the area in the subsequent night (Figure 4). Departure probability decreased with surface temperature (*temp.z*). Available tailwind component at midnight during the study period varied only little 1.8 ± 3.0 m s^-1^ (range: [-4.3 m s^-1^, 7.4 m s^-1^], *n* = 21) and did not significantly influence wheatears’ departure decision (Tables 2 and 3). Detection probability was higher in radio-tagged birds compared to colour-marked ones, but here surface wind speed (*surfwind.z*) did not influence birds’ detection probability (Table 3).

**Table 3:** Estimating staging and detection probabilities of northern wheatears with information about their evening fuel load

**Predictors**	**Mean**	**2.5%**	**97.5%**	**Rhat**	**n.eff**
Staging probability					
*intercept (α*_*1*_*)*	4500	3.053	5.980	1.002	6900
***daysa***_***i,t ***_***(α***_***2***_***)***	**-0**.**474**	**-0.799**	**-0.147**	**1.001**	**20000**
*day.c*_*t*_*(α*_*3*_*)*	-0.108	-0.308	0.091	1.001	3800
***temp.z***_***t ***_***(α***_***4***_***)***	**0.787**	**0.059**	**1.606**	**1.001**	**20000**
*wind.z*_*t*_*(α*_*5*_*)*	-0.118	-0.614	0.370	1.001	20000
*twc.z*_*t*_*(α*_*6*_*)*	0.236	-0.382	0.878	1.001	20000
*indfirst*_*i,t*_*(α*_*7*_*)*	-0.830	-2.251	0.734	1.001	20000
***efl***_***i,t ***_***(α***_***8***_***)***	**-3.915**	**-6.760**	**-1.098**	**1.001**	**20000**
*efl*_*i,t*_*x day.c*_*t*_*(α*_*9*_*)*	0328	-0.106	0.766	1.001	20000
Detection probability					
*intercept (β*_*1*_*)*	2.946	1.665	4.515	1.001	2000
***radio-tagged***_***i ***_***(β***_***2***_***)***	**30.796**	**4.328**	**64.840**	**1.001**	**2000**
*surfwind.z*_*t+1*_*(β*_*3*_*)*	-0.143	-0.929	0.754	1.001	3700
deviance	26.497	16.380	42.420	1.001	6100

As Table 2, but for model fitted in the second step using the 30 individuals with evening fuel load measurements in addition to the 75 individuals analysed in Table 2.

### Departure direction and departure time

For 16 wheatears we could identify departure times and, with the exception of one, their departure directions. Alaskan wheatears departed in a uniform direction towards 233° from Wales (Rayleigh test of uniformity: *n* = 15, *R* = 0.87, *P* < 0.0001; 95% CI: 218° – 249°, range: [205°, 288°]; Figure 2). This demonstrated that juvenile Alaskan wheatears did not followed the great circle route when setting off from Wales, as the corresponding 95% CI of their departure direction did not include the predicted direction of 336°. Predicted directions for the constant geographic course (251°) and the constant magnetic course (262°) were close to the mean departure direction (Figure 2). Corresponding mean sea barriers to be crossed were 176 km (± 98 km, range: [45 km, 265 km], *n* = 15) with 45 km to the Diomede Islands.

**Figure 5:**
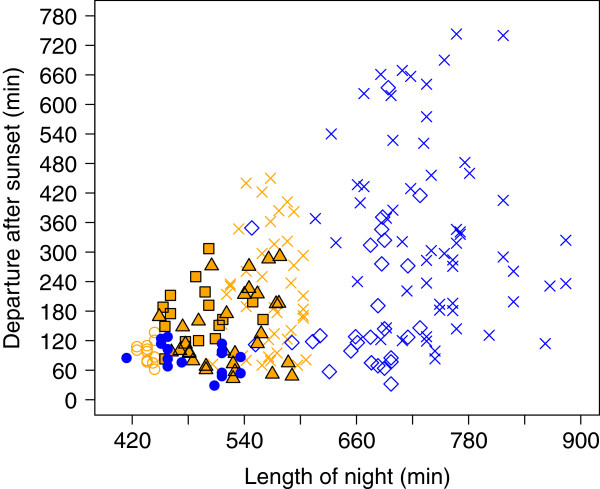
**Departure time of radio-tracked songbirds in relation to the length of the night. ** Blue = autumn, orange = spring migration. Blue circle: northern wheatears from Wales, Alaska, in autumn (this study); orange square edged black: northern wheatears of the *oenanthe* subspecies passing Helgoland, Germany, in spring [15]; orange triangle edged black: northern wheatears of the *leucorhoa* subspecies passing Helgoland, Germany, in spring [14]; orange ring: sedge warbler passing Rybachy, Russia, in spring [18]; blue diamond: reed warblers passing Falsterbo, Sweden, in autumn [17]; blue and orange crosses: Eurasian robins passing Rybachy Russia [16]. Northern wheatears at Wales did not set off at a lower proportion of night than sedge warblers in Rybachy (Wilcoxon test: *W *= 61, *P *= 0.11), but it differed in general significantly between the considered species (GLM with binomial error distribution: *P <*0.0001, Additional file 9).

Wheatears took off around 0:34 a.m. local time. Length of night, i.e., sunset to sunrise, increased from 410 to 554 min during the study. Wheatears took-off before the end of nautical twilight, when the sun was at maximum 9.5° below the horizon (sun’s elevation: -7.0 ± 1.7°, range: [-9.5°, -3.5°], *n* = 16), which was equivalent to 85 min (± 29 min, range: [29 min, 128 min]) after sunset (Figure 5). Neither sun’s elevation at departure, departure time after sunset, nor flight time within the night, sea barrier distance, nor departure direction correlated with departure fuel load (Spearman rank and circular-linear correlations: *P-values* > 0.24). Wheatears departed within the first 17.8 ± 6.6% (*n* = 16) of the night.

## Discussion

The weak correlation of departure fuel load and fuel deposition rate suggests that wheatears about to cross the Bering Strait did not behave according to a time minimizing strategy (Figure 3). That the birds carried considerable surplus fuel load at departure contradicts, however, that they strictly minimize the overall energy cost of transport (Figure 3; [1]). The day-to-day departure probability increased with evening fuel load indicating the influence of the birds’ physical condition on departure. By taking-off early in the night Alaskan wheatears maximized their potential nocturnal travel range. Departure directions towards southwest contradicted the predicted great circle route for Alaskan wheatears during autumn migration [27] and were more in line with the constant geographic and magnetic course [3].

### Fuel load and departure decisions

Juvenile wheatears arriving for the first time at a sea barrier stopped over for an average of 4 to 5 days, despite having sufficient fuel load for crossing the Bering Strait (Figure 1). Therefore, the Bering Strait cannot be seen as an ecological barrier for them, and wheatears had a free choice of where to stopover [4]. Surplus fuel load upon arrival is in agreement with evidence from the vast majority of migratory songbirds that hardly deplete their fuel reserves when migrating over hospitable land [33,65-67] or even across the Sahara desert [68-71]. However, land birds migrating long distances over water may arrive at stopover sites with strongly depleted reserves. These birds may behave differently at a stopover site than the juvenile wheatears arriving in good condition.

Generally, migrants are assumed to behave as time minimizers [5,10], as shown for wheatears of the Palaearctic-African flyway (Figure 3; [8,9]). In contrast, our results from Alaskan wheatears indicated an energy saving strategy (Figure 3). For time-minimizers daily fuel deposition rate should influence departure probability [13], but this could not be considered in our model. However, as evening fuel load contains the fuel gain over time depending on arrival day and arrival fuel load, the observed increase in departure probability with evening fuel load (Figure 4) is related to what is expected for the minimization of time and minimization of total energy cost of migration [1]. Furthermore, Alaskan wheatears departed with three- to five-times higher fuel loads than assumed for a 9 h flight corresponding to the longest night during the study (Figure 3). This considerable surplus enables wheatears to by-pass potential stopover sites en route and thus speed up their migration stages, which is considered typical for time-minimizers [72]. Alaskan wheatears being tracked by light-level geolocators did so by migrating on average 4,900 km without staying more than two days at the same spot after leaving Alaska or Chukotka [3]. Hence, there is ample evidence that wheatears have the capability of skipping stopover sites en route as also shown for other migrants [73-75]. Furthermore, the Russian Far East and Siberian taiga belt may offer only small and infrequent patches of open habitat suitable for wheatears to refuel, as shown for Bluethroats (*Luscinia luscinia*) [76]. It may be, hence, advantageous for wheatears to have a certain fuel load prior leaving Alaska. Carrying such extra fuel load increases the overall energy cost of transport which rejects this strategy for our wheatears [1]. We conclude that the general trade-off between minimizing energy or time of migration may be superimposed by a third aspect, i.e., fuel safety: In line with speculations on waders [72], surplus fuel load may enable migrants to withstand unpredictable adverse condition at future sites en route.

In contrast to other studies [12,14,46,53,77,78], wind did not play a significant role for our birds. Variation and strongest headwind (4.3 m s^-1^) were probably too low to influence departure probability of wheatears, because only headwinds > 7 m s^-1^ are supposed to be unfavourable for migration [46]. Wheatear’s departure probability increased with decreasing temperature (Figure 4). This may be a reaction to the increase in energy costs on the ground with decreasing temperature [2,3]. Changes in temperature may also coincide with a shift in pressure system and wind conditions [12], but the daily tailwind component did not change with temperature nor with season (Wilcoxon-tests: *P-values* > 0.17, *n* = 21).

It needs to be considered that supplementary feeding is influencing the behavior of the birds. Nevertheless, baited balances are the most efficient and common method to estimate departure fuel load [7-9,30-37]. On the island Helgoland (Germany) food, i.e., kelp fly larvae (Coelopidae), is regularly superabundant when kelp algae are washed onshore. Under such natural circumstances offered mealworms are rejected and fuel load of wheatears can be extremely high, i.e., 130% of bird’s lean body mass (cf. Figure 3; V. Dierschke pers. comm.; [79,80]). Hence, fuel loads recorded with baited balances cannot be considered generally higher than under natural conditions [7,8,67,80], but see [34,81].

### Departure direction and departure time

The south-westerly departure directions of our juvenile wheatears were in agreement with overall migration directions of the three Alaskan being tracked with light-level geolocators when leaving Alaska [3]. This demonstrates that, in contrast to earlier hypotheses [27], inexperienced and experienced Alaskan wheatears did not follow the “great circle route” towards their wintering area [3]. The observed variation in departure directions was in agreement with the predicted directions of the constant geographic and constant magnetic course. However, considering the entire migration route of the tracked Alaskan wheatears demonstrated that they followed neither compass course all along to their wintering area [3], as also shown for other Arctic migrants [49].

Alaskan wheatears took off within a small time window shortly after sunset, i.e., before the end of nautical twilight when skylight polarization pattern may be used for calibrating the compass systems [19,82-84]. Our birds departed at higher sun elevations and earlier in relation to the proportion of the night as compared to other studies (GLM with binomial error distribution: *P <* 0.0001; Figure 5 and Additional file 9; [14-18]). Alaskan wheatears took off earlier in the night than in two studies of wheatears departing from Helgoland in spring (Wilcoxon tests: *P-values* < 0.001) (Additional file 9). The simple rule may be to take-off early, when nights are short. This overall pattern is modified by the effect of body condition on departure time [14]. We hypothesize that nocturnal migrants consider internal information (e.g. body condition) jointly with the current night length, i.e., the available traveling time (external information), for their departure decision within the night.

## Conclusions

As Alaskan wheatears migrate about 30,000 km during six months each year [3], their general costs (including risk of predation and for foraging) and energy costs of migration are likely to be higher than in other life history stages [1]. If so, migration can be regarded as an energetic bottleneck. Minimizing the total energy cost of migration might be, therefore, in favour of selection [1], enabling wheatears to optimize energy and time spent on migration.

Departure time in combination with wind conditions defines the potential nocturnal travel range, which influences the total number of stopovers required. Therefore, an early timing of nocturnal flight stages may considerably speed up the overall migration and affect the energy costs of migration. Although Alaskan wheatears did not behave as strict time-minimizers (Figure 3), exploiting the entire night for migration may significantly minimize the time of migration, in particular if this is done consistently throughout migration [85]. Our results provide explanations for the high nocturnal travel speed of 330 km per night across 15,000 km and the average nocturnal flight duration of 7 h found in Alaskan wheatears [3]. Therefore, we argue that nocturnal departure time is a crucial factor shaping speed of migration. Given the long migration distance in Alaskan wheatears, variation in the timing of take-off and the total time available for single nocturnal flight stages may strongly affect the overall time and energy costs per migration cycle.

## Competing interests

The authors declare that they have no competing interests.

## Authors’ contributions

HS and FB proposed the research idea. HS designed the research, carried out the field work, wrote the manuscript, analysed all data except the mark-recapture model and provided Table 1, Figures 1, 2, 3, 5 and the Additional files 1, 2, 7, 8, 9. FK-N calculated the mark-recapture model for estimating departure probability and stopover duration, prepared Tables 2 and 3, Figure 4 and produced the Additional files 3, 4, 5, 6. BN-D wrote parts about the radio tracking paragraph and supported the radio tracking study. RN, IM and MB carried out field work. All authors contributed to drafting and critically revising the manuscript and have read and approved the final version of the manuscript.

## Supplementary Material

Additional file 1 Optimal migration strategy, documentation. Click here for file

Additional file 2 Radio tracking, documentation. Click here for file

Additional file 3 Modelling evening fuel load, documentation. Click here for file

Additional file 4 Evening fuel load over day since arrival for 30 northern wheatears, figure. Click here for file

Additional file 5 Markov chain Monte Carlo simulations to fit mark-recapture model, documentation. Click here for file

Additional file 6 Monte Carlo simulations estimating stopover duration, documentation. Click here for file

Additional file 7 Relative abundance of northern wheatears, figure. Click here for file

Additional file 8: Individual data of wheatears at initial capture, table. Click here for file

Additional file 9 Sun’s elevation at departure of radio-tracked songbirds and their departure time after sunset, figure. Click here for file
