# Supplementary material for: Stopover optimization in a long-distance migrant: the role of fuel load and nocturnal take-off time in Alaskan northern wheatears (Oenanthe oenanthe)
Source: Front Zool. 2013 May 12;10:26. doi: 10.1186/1742-9994-10-26 (PMC3665591; doi:10.1186/1742-9994-10-26)
Supplement: Additional file 1 — Optimal migration strategy, documentation. [file 1742-9994-10-26-S1.pdf]

Additional file 1

Optimal bird migration strategies

We used wheatears' fuel deposition rate and departure fuel load to discern their optimal migration strategy [1,2]: (1) Minimization of time, i.e., maximizing speed of migration by selecting stopovers with high fuel deposition rate yielding a positive correlation of departure fuel load and fuel deposition rate. (2): Minimization of the overall energy costs of transport by departing from a stopover site with just as much fuel as required for the next flight stage. Departure decision is independent of fuel deposition rate and stopover duration as all birds are supposed to resume migration with the same amount of fuel, i.e., lacking a correlation of departure fuel load and fuel deposition rate. (3): Minimization of the total energy costs of migration showing an intermediate behaviour between the former and the latter by minimizing stopover duration and avoiding high fuel loads.

References

1. Alerstam T, Lindström Å: **Optimal bird migration: the relative importance of time, energy, and safety.** In *Bird Migration: Physiology and Ecophysiology*. Edited by Gwinner E. Berlin Heidelberg: Springer; 1990:331-351.
2. Hedenström A, Alerstam T: **Optimum fuel loads in migratory birds: Distinguishing between time and energy minimization.** *J Theor Biol* 1997, **189:**227-234.
