# Supplementary material for: Stopover optimization in a long-distance migrant: the role of fuel load and nocturnal take-off time in Alaskan northern wheatears (Oenanthe oenanthe)
Source: Front Zool. 2013 May 12;10:26. doi: 10.1186/1742-9994-10-26 (PMC3665591; doi:10.1186/1742-9994-10-26)
Supplement: Additional file 2 — Radio tracking, documentation. [file 1742-9994-10-26-S2.pdf]

Additional file 2

Radio tracking

The birds were located by triangulation from subsequent observer positions. During each departure event birds were radio tracked from an elevated position (1–2 km inland from the coast) and from the cost line, where one observer was stationed for most of the times. The bearing accuracy, as the average deviation from true azimuth, was 3° (SD 5°, $n = 49$) in a former study [1]. The area on the ground covered by us during the nocturnal tracking survey was about 2 x 2 km^2. Hence, a potential parallax error in direction estimates was small compared to the bearing accuracy of hand-held antennas [2]. Set-off distance between bird and observer was less than 500 m. The corresponding parallax error in respect to a tracking distance of 15 km would be < 2°. For further information about the radio tracking method see [1,3].

References

1. Schmaljohann H, Becker PJJ, Karaardic H, Liechti F, Naef-Daenzer B, Grande C: **Nocturnal exploratory flights, departure time, and direction in a migratory songbird.** *J Ornithol* 2011, **152:**439-452.

2. Kenward RE: *A Manual for Wildlife Radio Tagging*. London, San Diego: Academic Press; 2001.

3. Schmaljohann H, Naef-Daenzer B: **Body condition and wind support initiate shift in migratory direction and timing of nocturnal departure in a free flying songbird.** *J Anim Ecol* 2011, **80:**1115-1122.
