# Supplementary material for: Stopover optimization in a long-distance migrant: the role of fuel load and nocturnal take-off time in Alaskan northern wheatears (Oenanthe oenanthe)
Source: Front Zool. 2013 May 12;10:26. doi: 10.1186/1742-9994-10-26 (PMC3665591; doi:10.1186/1742-9994-10-26)
Supplement: Additional file 3 — Modelling evening fuel load, documentation. [file 1742-9994-10-26-S3.pdf]

Additional file 3

Modelling evening fuel load

Each evening fuel load could not be measured for every of the 30 birds, because not all present birds fed within two hours before sunset (Additional file 4). Evening fuel load was measured only once in one bird, but usually two to four times per individual (median = 3). Thus, the variable evening fuel load ($efl_{i,t}$) contained missing values. These missing values were imputed by using a linear mixed regression on day since arrival ($daysa_{i,t}$) with individual intercept and individual slope:

$efl_{i,t} \sim \mathrm{Norm}(mefl_{i,t}, \sigma_{efl})$

$mefl_{i,t} = \gamma_0 + b_i + (\gamma_1 + d_i)daysa_{i,t}$

with $b_i \sim \mathrm{Norm}(0, \sigma_b)$ and $d_i \sim \mathrm{Norm}(0, \sigma_d)$.

We then used the model predictions for each individual and day for those days where no measurements were available.
