# Supplementary material for: Stopover optimization in a long-distance migrant: the role of fuel load and nocturnal take-off time in Alaskan northern wheatears (Oenanthe oenanthe)
Source: Front Zool. 2013 May 12;10:26. doi: 10.1186/1742-9994-10-26 (PMC3665591; doi:10.1186/1742-9994-10-26)

Additional file 4

Figure Evening fuel load over day since arrival for 30 northern wheatears. Bold line and grey area = Regression line with 95% CrI for the population (mean over all 30 individuals), blue dotted lines = individual specific regression lines (from the mixed linear model), circles = measurements. Measurements of the same individual are connected by black lines.

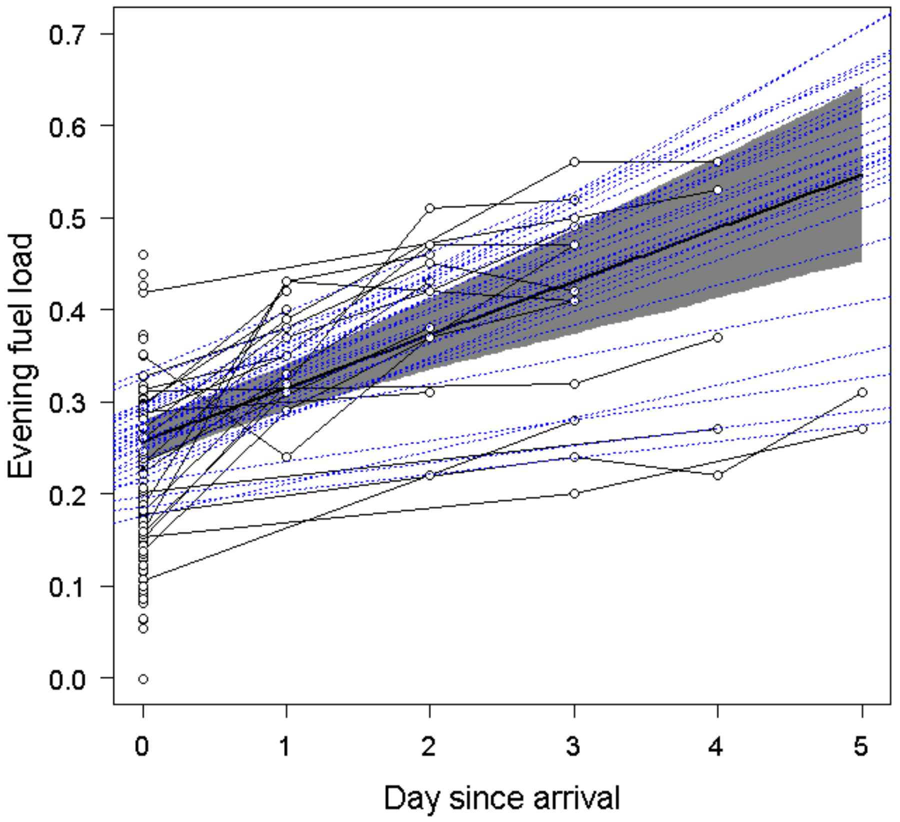

Supplement: Additional file 4 — Evening fuel load over day since arrival for 30 northern wheatears, figure. [file 1742-9994-10-26-S4.pdf]
