# Supplementary material for: Stopover optimization in a long-distance migrant: the role of fuel load and nocturnal take-off time in Alaskan northern wheatears (Oenanthe oenanthe)
Source: Front Zool. 2013 May 12;10:26. doi: 10.1186/1742-9994-10-26 (PMC3665591; doi:10.1186/1742-9994-10-26)
Supplement: Additional file 5 — Markov chain Monte Carlo simulations to fit mark-recapture model, documentation. [file 1742-9994-10-26-S5.pdf]

Additional file 5

Markov chain Monte Carlo simulations to fit mark-recapture model

We applied Markov chain Monte Carlo simulations performed in WinBUGS to fit the mark-recapture models [1]. Three chains were run, each of length 50000. The burn-in was set to 5000 and each 5th element was used to describe the posterior distributions of the model parameters. WinBUGS was started via the R-interface R2WinBugs [2], and all other data handling and data analyses were done in R 2.13. 2 [3].

References

1. Spiegelhalter D, Thomas A, Best N: *WinBUGS User Manual, Version 1.2*. Cambridge: MCR Biostatistics Unit; 2003.

2. Sturtz S, Ligges U, Gelman A: **R2WinBUGS: A package for running WinBUGS from R.** *J Stat Softw* 2005, **12:**1-16.

3. R Development Core Team. R: a language and environment for statistical computing. 2011. Vienna, R Foundation for Statistical Computing. http://www.R-project.org.
