# Supplementary material for: Stopover optimization in a long-distance migrant: the role of fuel load and nocturnal take-off time in Alaskan northern wheatears (Oenanthe oenanthe)
Source: Front Zool. 2013 May 12;10:26. doi: 10.1186/1742-9994-10-26 (PMC3665591; doi:10.1186/1742-9994-10-26)
Supplement: Additional file 6 — Monte Carlo simulations estimating stopover duration, documentation. [file 1742-9994-10-26-S6.pdf]

Additional file 6

Monte Carlo simulations estimating stopover duration

The Monte Carlo simulation was done for each individual separately and included the following iterations:

1) Evening fuel load was predicted by the linear mixed regression [see Additional files 3 and 4] for all possible days since arrival. Specifically, we did this until day 100 since arrival which was clearly above the maximum stopover duration.

2) For each day since arrival t the staging probability Φ^*_t was obtained as the inverse logistic function of the linear predictor from the second mark-recapture model. Temperature, wind, and tailwind components for the hypothetical days after day 21, which were beyond the study period and no data was available, were assumed to be the means of the study period.

3) A stopover of a bird was simulated by the autoregressive Bernoulli process:

$z^*_1 = 1$

$z^*_{t+1} \sim Bernoulli(z^*_t \Phi^*_{t-1})$

4) The sum $\sum_{t=1}^{100} z_t^*$ is one simulation of the stopover duration for the specific individual.

5) Iterations 3 and 4 were repeated 2000 times. The mean, standard deviation, 2·5% and 97·5% quantiles of the 2000 simulated stopover durations were used as estimate, standard error and 95% prediction interval for the stopover duration of the specific individual.

Estimated stopover durations included the day of trapping.
