# Supplementary material for: Stopover optimization in a long-distance migrant: the role of fuel load and nocturnal take-off time in Alaskan northern wheatears (Oenanthe oenanthe)
Source: Front Zool. 2013 May 12;10:26. doi: 10.1186/1742-9994-10-26 (PMC3665591; doi:10.1186/1742-9994-10-26)

Additional file 7

Figure Relative abundance of northern wheatears trapped (black bars, *n* = 105) and observed (grey dots, *n* = 1535) around the study site in Wales over time of season.

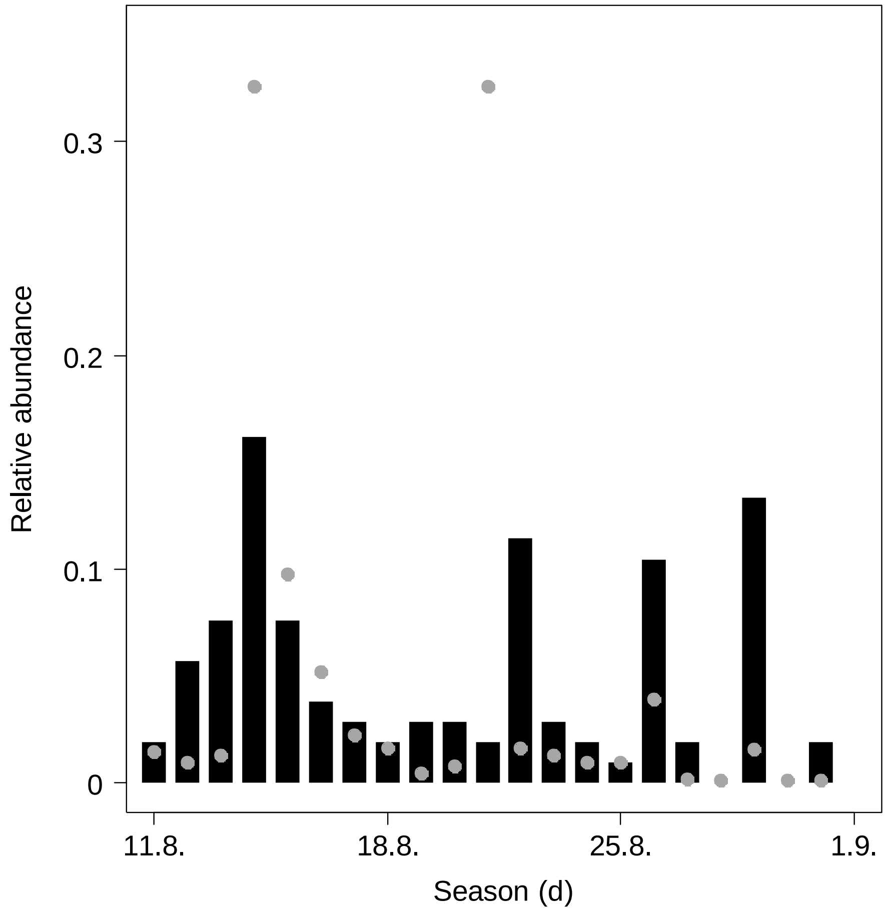

Supplement: Additional file 7 — Relative abundance of northern wheatears, figure. [file 1742-9994-10-26-S7.pdf]
