# Supplementary material for: Stopover optimization in a long-distance migrant: the role of fuel load and nocturnal take-off time in Alaskan northern wheatears (Oenanthe oenanthe)
Source: Front Zool. 2013 May 12;10:26. doi: 10.1186/1742-9994-10-26 (PMC3665591; doi:10.1186/1742-9994-10-26)
Supplement: Additional file 8: — Individual data of wheatears at initial capture, table. [file 1742-9994-10-26-S8.pdf]

Additional file 8

Table. Individual data of ringed northern wheatears. Date and local time of capture, body weight, fat score [1], muscle score [2], tarsus length, and maximum wing length [3] at initial capture.

Ind. [ring number]	Date and time of capture	Body weight [g]	Fat score	Muscle score	Tarsus length	Wing length
179106208	11.08.2010 19:50	32	4	2	26.7	99.2
179106209	12.08.2010 18:50	26.7	2	2	26.4	100.2
179106210	12.08.2010 18:50	24.7	2	2	26	97.5
179106211	12.08.2010 18:50	30.2	4	2	27.4	103.5
179106212	12.08.2010 18:50	31.2	4	3	26.9	99.5
179106213	12.08.2010 18:50	25.7	2	2	27.5	102.7
179106214	12.08.2010 18:50	30	4	2	26.6	102.5
179106215	13.08.2010 18:00	31	4	2	26	101.5
179106216	13.08.2010 18:00	27.5	3	3	26.8	100.5
179106217	13.08.2010 18:00	25	2	2	27.7	97.2
179106218	13.08.2010 19:00	28.5	4	3	27.2	99.7
179106219	13.08.2010 19:00	26	2	2	27.7	104.7
179106220	13.08.2010 19:00	24	2	2	27	97
179106221	13.08.2010 19:00	28	4	2	27	98.5
179106222	13.08.2010 19:00	26.5	3	3	27.5	103.5
179106223	14.08.2010 14:00	27.5	2	2	27.2	100.5
179106224	14.08.2010 14:00	29.2	4	2	28	99.5
179106225	14.08.2010 14:05	24.7	2	1	26.1	101.7
179106226	14.08.2010 14:00	30.5	4	3	26.8	100.5
179106227	14.08.2010 14:15	26.5	2	2	27.4	103.5
179106228	14.08.2010 14:15	25.2	2	2	27.9	98.7
179106229	14.08.2010 14:20	26.5	3	3	27.4	101.2
179106230	14.08.2010 14:30	26.2	2	1	28.1	103
179106231	14.08.2010 14:30	26.5	4	2	26.2	98
179106232	14.08.2010 14:30	29.2	4	2	25.6	98.2
179106233	14.08.2010 14:45	27	3	2	27.5	99
179106234	14.08.2010 15:00	25.5	1	2	26.5	100.2
179106235	14.08.2010 15:00	27.7	3	2	27.7	101
179106236	14.08.2010 15:00	33	4	3	26.6	102.7
179106237	14.08.2010 15:15	27.5	4	2	26.2	96
179106238	14.08.2010 18:45	29.2	4	3	26.6	100.2
179106239	14.08.2010 18:45	30.2	4	2	26.8	102
179106240	15.08.2010 13:20	27.7	2	2	27.6	101.7
179106241	15.08.2010 13:20	28	3	2	27.6	98.7
179106242	15.08.2010 13:20	29.2	2	2	27.2	103.5

Additional file 8

179106243	15.08.2010 13:45	25.5	2	2	26.8	100.5
179106244	15.08.2010 14:00	27.7	2	2	27.5	100.7
179106245	15.08.2010 15:15	25.5	3	2	25.7	99
179106246	15.08.2010 15:15	29	3	2	27.6	99.5
179106247	15.08.2010 18:17	27.5	3	3	26.6	100.5
179106248	16.08.2010 11:35	24	2	2	27.8	102.2
179106249	16.08.2010 11:35	24.7	2	2	27.7	100
179106250	16.08.2010 13:45	32	3	2	27.7	101
179106251	16.08.2010 19:30	24.2	2	2	26.9	102
179106252	17.08.2010 13:30	29.5	2	1	28.3	101.7
179106253	17.08.2010 13:30	28.2	3	2	27.5	98.5
179106254	17.08.2010 14:20	27.5	3	2	26.9	104.5
179106255	18.08.2010 13:00	25.5	2	2	26.1	101.2
179106256	18.08.2010 14:00	24	2	2	27.1	100.2
179106257	19.08.2010 16:15	24.2	2	2	26.1	100.2
179106258	19.08.2010 16:15	26	2	2	28.3	103
179106259	19.08.2010 16:20	24.2	2	2	27.3	100.5
179106260	20.08.2010 14:30	23.7	1	1	27.2	100.5
179106261	20.08.2010 14:35	27	2	2	28.3	101.5
179106262	20.08.2010 22:10	26.2	2	2	25.9	102
179106263	21.08.2010 14:30	25.5	2	2	27	103.2
179106264	21.08.2010 17:25	28.5	2	2	28.2	101
179106265	22.08.2010 14:45	25.7	2	2	25.6	97.5
179106266	22.08.2010 14:45	26.2	2	2	27.3	101.7
179106267	22.08.2010 15:00	27.5	2	1	26.3	103.5
179106268	22.08.2010 15:00	26	3	2	25.9	97.5
179106269	22.08.2010 15:00	25.2	2	2	27.3	101.5
179106270	22.08.2010 15:10	26	2	1	26.3	103
179106271	22.08.2010 15:10	28.7	2	1	26.7	103.5
179106272	22.08.2010 15:15	30.5	2	2	27.8	104.7
179106273	22.08.2010 15:20	30.7	3	2	27.7	104.2
179106274	22.08.2010 15:20	26.5	2	2	27.6	103
179106275	22.08.2010 15:30	31	4	2	27.3	102.7
179106276	22.08.2010 15:45	28.2	3	2	26.6	99.5
179106277	23.08.2010 14:15	25	2	2	28	98
179106278	23.08.2010 14:15	29.5	3	2	27.5	102.2
179106279	23.08.2010 14:15	26	1	2	27.3	101.2
179106280	24.08.2010 15:45	27.7	3	2	26.8	104.5
179106281	24.08.2010 16:00	25.2	3	2	27.5	103
179106282	25.08.2010 14:00	25.2	3	2	27.2	96
179106283	26.08.2010 14:10	28.7	4	2	27.2	103.2

Additional file 8

179106284	26.08.2010 14:15	26	2	2	28	100.2
179106285	26.08.2010 14:30	26.2	4	3	27.5	98.5
179106286	26.08.2010 14:30	29	3	2	27.5	107.5
179106287	26.08.2010 14:30	25.7	3	2	26.7	99.7
179106288	26.08.2010 14:40	27.2	3	2	25.9	102
179106289	26.08.2010 15:20	27	3	2	26.3	99.7
179106290	26.08.2010 16:40	27.2	4	2	27.3	98
179106291	26.08.2010 16:55	25.5	2	2	26.4	103
179106292	26.08.2010 16:55	25	2	2	28	102
179106293	26.08.2010 16:55	30	3	2	27.7	105
179106294	27.08.2010 16:10	24.5	1	2	28.2	103
179106295	27.08.2010 16:10	21.7	2	2	25.7	98.5
179106296	29.08.2010 10:10	25.5	3	2	26.4	100.5
179106297	29.08.2010 10:10	28.5	3	2	26.6	102.5
179106298	29.08.2010 10:10	26.2	3	2	27	103.2
179106299	29.08.2010 13:35	24.2	3	3	25.3	93.7
179106300	29.08.2010 13:35	25	3	3	26.9	98
179106301	29.08.2010 14:15	26.7	3	2	26.7	101.5
179106302	29.08.2010 14:15	28	2	2	28.2	99
179106303	29.08.2010 14:35	26	2	2	27.4	103.5
179106304	29.08.2010 15:00	26	3	2	26.3	97
179106305	29.08.2010 15:00	28	2	3	27.7	99.5
179106306	29.08.2010 15:00	25.2	2	2	27.4	100
179106307	29.08.2010 15:20	26	2	2	27.8	98.5
179106308	29.08.2010 15:25	26	2	2	26.4	98.7
179106309	29.08.2010 15:30	27	3	3	26.2	102
179106310	31.08.2010 12:00	24.5	2	2	26	103
179106311	31.08.2010 12:05	25.5	3	3	25.3	98

References

1. Kaiser A: **A new multi-category classification of subcutaneous fat deposits of songbirds**. *J Field Ornithol* 1993. **64**:246-255.

2. Bairlein F: *Manual of Field Methods. European-African Songbird Migration*. Wilhelmshaven: Institut für Vogelforschung; 1994.

3. Svensson L: *Identification guide to European passerines*. 4th edn. Stockholm: BTO; 1992.
