# Supplementary material for: Stopover optimization in a long-distance migrant: the role of fuel load and nocturnal take-off time in Alaskan northern wheatears (Oenanthe oenanthe)
Source: Front Zool. 2013 May 12;10:26. doi: 10.1186/1742-9994-10-26 (PMC3665591; doi:10.1186/1742-9994-10-26)
Supplement: Additional file 9 — Sun’s elevation at departure of radio-tracked songbirds and their departure time after sunset, figure. [file 1742-9994-10-26-S9.pdf]

Additional file 9

Figure Sun's elevation at departure of radio-tracked songbirds (a) and their departure time after sunset in relation to length of night (b). Black = autumn, grey = spring migration. Data is shown for northern wheatears (NW) from Wales (Alaska), northern wheatears of the *oenanthe* subspecies from Helgoland (Germany) [1] and the *leucorhoa* subspecies from Helgoland (Germany) [2], sedge warblers (*Acrocephalus schoenobaenus*, SW) from Rybachy (Russia) [3], reed warblers (*Acrocephalus scirpaceus*, RW) from Falsterbo (Sweden) [4] and Eurasian robins (*Erithacus rubecula*, ER) from Rybachy (Russia) [5]. Corresponding sample size is given below the boxes. **: $P < 0{\cdot}01$.

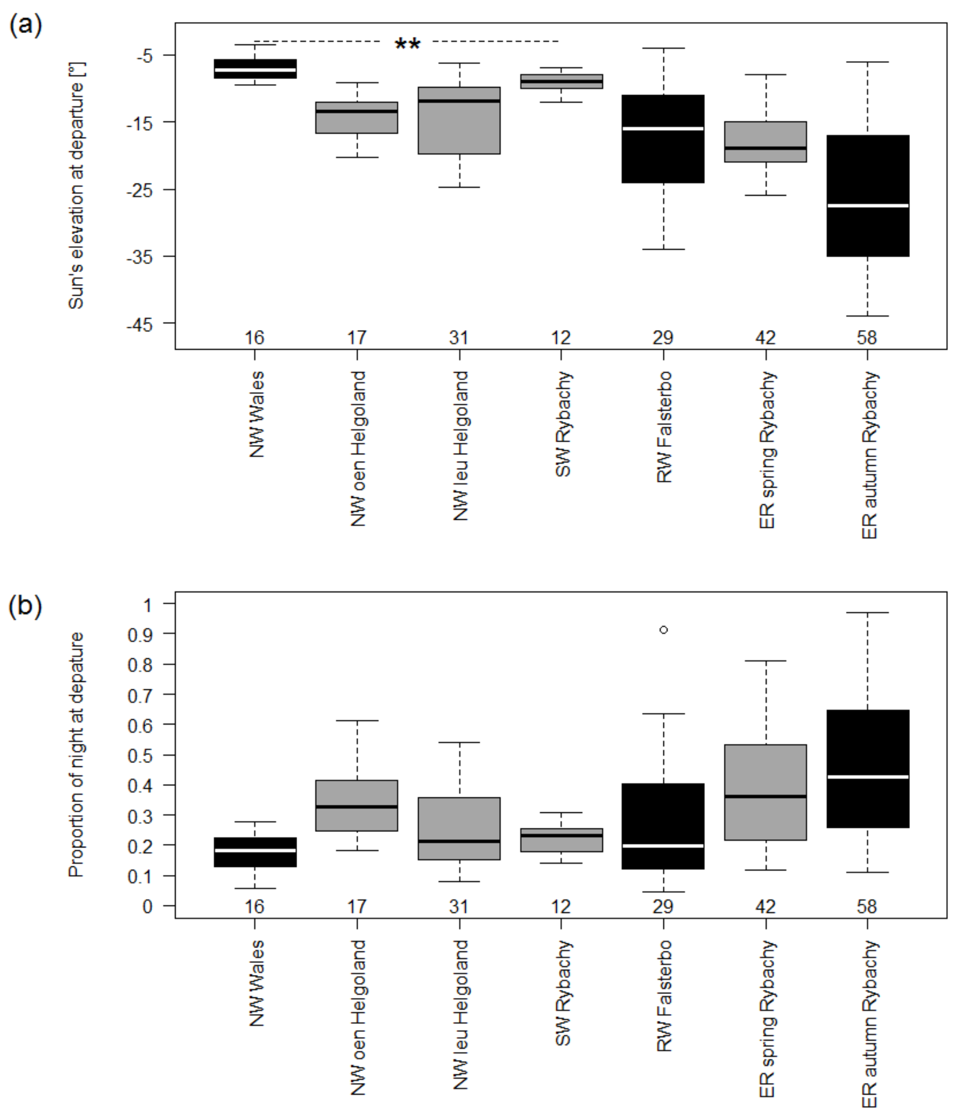

Additional file 9

References

1. Schmaljohann H, Becker PJJ, Karaardic H, Liechti F, Naef-Daenzer B, Grande C: **Nocturnal exploratory flights, departure time, and direction in a migratory songbird.** *J Ornithol* 2011,**152:** 439-452.

2. Schmaljohann H, Naef-Daenzer B: **Body condition and wind support initiate shift in migratory direction and timing of nocturnal departure in a free flying songbird.** *J Anim Ecol* 2011, **80:**1115-1122.

3. Bolshakov CV, Chernetsov N: **Initiation of nocturnal flight in two species of long-distance migrants (*Ficedula hypoleuca* and *Acrocephalus schoenobaenus)* in spring: a telemetry study.** *Avian Ecol Behav* 2004,**12:** 63-76.

4. Åkesson S, Walinder G, Karlsson L, Ehnbom S: **Reed warbler orientation: initiation of nocturnal migratory flights in relation to visibility of celestial cues at dusk.** *Anim Behav* 2001, **61:**181-189.

5. Bolshakov CV, Chernetsov N, Mukhin A, Bulyuk V, Kosarev VV, Ktitorov P *et al*.: **Time of nocturnal departures in European robins, *Erithacus rubecula,* in relation to celestial cues, season, stopover duration and fat score.** *Anim Behav* 2007,**74:** 855-865.
